# Physical health conditions and political participation in Europe: the moderating effects of age

**DOI:** 10.1057/s41295-023-00347-3

**Published:** 2023-05-07

**Authors:** Andrej Kirbiš, Mikko Mattila, Lauri Rapeli

**Affiliations:** 1 Faculty of Arts, University of Maribor, Koroška cesta 160, 2000 Maribor, Slovenia; 2 Political Science, University of Helsinki, PO Box 54 (Unioninkatu 37), 00014 Helsinki, Finland; 3 Åbo Akademi University, ASA A4, 20500 Turku, Finland

**Keywords:** Health, Political participation, Youth, Age, Physical health, Turnout

## Abstract

Unequal political participation is widely considered a problem of democratic representation. Citizens with fewer resources typically report lower levels of participation. Lack of good health has been identified as one barrier to participation. However, poor health may have heterogeneous impacts on participation, depending on the type of health issue. Moreover, poor health may affect participation patterns differently, depending on age. Previous research has not yet systematically examined these issues. We address these gaps by using European Social Survey data, which includes self-reports of a variety of physical health conditions and engagement in different forms of political participation. The results show that most physical health conditions are related to political participation; however, except for turnout, physical health problems mobilize individuals into action. This effect is strongest among younger individuals, and the health gap in participation evens out in later life. The condition-specific effects are similar across different forms of physical health conditions. Our findings are consistent with the grievance and identity theories of political participation. Younger citizens, in particular, may experience poor health or physical impairment as unjust and are then mobilized into political action. We discuss the implications for the broader understanding of mechanisms behind political behavior and suggest that health problems are often a motivator for political action rather than an obstacle.

## Introduction

Political participation—activities where citizens influence political decision-making—is a key condition for a democratic society. However, patterns of political participation are largely determined by various factors that are unequally distributed across society. There is strong evidence that citizens with fewer resources, including civic skills and fewer motivational, cultural, and social network resources, report lower levels of political participation (Norris 2002; Verba et al. 1995, 2003). In recent years, scholars have also increasingly acknowledged health as an important resource, the lack of which may hinder political participation (Mattila et al. 2018; Ojeda 2015). However, poor health may have a differential impact on political participation, depending on the type of health issue an individual faces and the barrier to participation it may represent. For example, while poor self-rated health (SRH) is associated with lower voter turnout, some health problems, such as cancer and asthma, were found to positively affect turnout (Gollust and Rahn 2015; Sund et al. 2017). Hence, poor health status may decrease some types of participation, e.g., voter turnout, but may *increase other forms of participation* (Couture and Breux 2017; Mattila 2020; Ojeda 2015; Söderlund and Rapeli 2015; Stockemer and Rapp 2019).

Additionally, previous scholarship has not adequately examined whether the impact of health on political participation is moderated by age. The existing studies assume, at least implicitly, that the potential effects of health issues remain constant over a person’s lifespan. However, it seems plausible that experiencing chronic illnesses in early adulthood is likely to have different consequences for political behavior patterns than experiencing similar issues in old age. There is some empirical evidence suggesting that poor health has a particular influence on turnout among older people (e.g., Mattila et al. 2013). However, systematic in-depth analyses are still lacking in the literature regarding various health conditions and different forms of participation at distinct stages of individuals’ lives.

Hence, we seek to make several original contributions to the literature on the relationship between health and participation. First, we examine various indicators of physical health conditions to answer how people are associated with various forms of political participation. Second, we address whether physical health negatively influences some forms of political participation more than others and whether some health conditions mobilize people into political action. Third, we examine whether age moderates the impact of health on political participation and, if so, in what direction. We use the seventh round of the European Social Survey (2015), which offers nationally representative samples of the adult population from 20 countries and includes the required health and participation measures.

## Various health conditions and political participation

Recently, *health status* has been established as an important but often overlooked resource that affects political participation (Couture and Breux 2017; Mattila et al. 2013; Söderlund and Rapeli 2015). Health status is especially relevant as a potential determinant of political behavior because health disparities may translate into “relevant political divisions that affect electoral outcomes and eventually public policy” (Pacheco and Fletcher 2015: 113), since individuals in poor health are likely to be underrepresented in the political process compared to their healthy counterparts (Ojeda and Pacheco 2019). In this article, we use the concept of physical health to refer to specific health conditions that respondents indicated having in the ESS survey. Although the terms are related, it is important to make a difference between a health condition and a disability. Chronic health conditions are often counted as disabilities, but on the other hand, many disabled people have excellent health. However, people with disabilities often face similar difficulties in their participation in political life as people with a chronic condition. Hence, the disability literature can, at least partly, guide our research (e.g., Schur and Kruse 2000; Schur et al. 2013; Powell and Johnson 2019; Reher 2020).

The link between health and political participation has been approached with different types of health variables to measure different facets of health conditions. Typical approaches have used survey-based measures of self-rated general health, specific physical or mental health conditions (or symptoms) and physical limitations. However, other kinds of data, such as official register data on sick days or medication purchases, have also been used (Sund et al. 2017; Lahtinen et al. 2017). Poorer general health has consistently been found to decrease voter turnout (Brown et al. 2020), as does poor mental health (Bernardi et al. 2022; Denny and Doyle 2007; Landwehr and Ojeda 2021). However, while most studies show that poor general health decreases turnout, studies of specific health conditions indicate a far less consistent picture. For example, poor SRH is associated with lower voter turnout, yet health conditions such as cancer and asthma (Sund et al. 2017) and long-term chronic conditions (Mattila et al. 2018; Stockemer and Rapp 2019) have been found to have a *positive* effect on turnout. Relatedly, studies on physical limitations have shown how voter turnout is affected by poor mobility (e.g., Schur and Kruse 2000). However, this effect may be related to age; in a longitudinal study of American youth, Ojeda and Pacheco (2019) found that poor self-rated health reduced the probability of voting, while physical limitations did not have a statistically significant effect on turnout.

Expanding the repertoire of health measures to include specific health conditions is important, since most previous studies focus only on SRH. According to Pacheco’s study (2019: 534), two-thirds of all the studies published on health and political behavior used SHR to measure health. However, when Pacheco used anchoring vignettes to adjust the SRH measure to account for interpersonal comparability, the statistically significant relationship between health and turnout vanished. Hence, while SRH is usually considered to be a valid and reliable indicator of one’s general health (DeSalvo et al. 2006; Idler and Benyamini 1997), examination of specific health issues is needed to provide a more nuanced understanding of which health conditions present the largest obstacles to political participation and which types of conditions might facilitate at least some forms of participation.

## Health and various forms of political participation

Similar to the predominant focus on SRH and self-reported chronic conditions as the main predictor variables in previous studies on health-related inequalities in political participation, the existing literature has also focused on one political outcome variable, namely turnout (Mattila et al. 2018). Studies consistently show poorer health is associated with lower voter turnout (Brown et al. 2020), which makes sense since health can be considered a resource for political action (Mattila et al. 2018) in the same way as other resources theorized by Verba et al. (1995) as relevant determinants of participation.

However, the handful of studies that have looked at other forms of participation besides voting has found that the impact of health is not identical across all types of political engagement. According to Burden et al. (2017), contributing money to political parties or campaigns did not vary depending on health status. Furthermore, some non-electoral forms of participation are higher among those with poorer health. Those with poorer SRH are more likely to sign petitions and initiatives (Christensen et al. 2019), and so are those with poorer mental health (Couture and Breux 2017). In addition, contacting politicians and participating in demonstrations are also more frequent among those with poorer health (Adman 2020; Mattila and Papageorgiou, 2017; Söderlund and Rapeli 2015). These findings indicate a “reversed health gap” (Söderlund and Rapeli 2015); for example, in Nordic countries, those with poorer health are more politically engaged through certain political activities than their healthier counterparts. Söderlund and Rapeli (2015) interpret this finding in two ways. First, easily accessible and convenient forms of political participation (e.g., wearing a political badge) are understandably more attractive to those with poorer health. Second, owing to the higher stakes for those in poorer health – being more dependent on the public sector for support – members of this group are also more likely to engage actively in more time-consuming political acts of influencing policymaking.

In terms of poor health, people may suffer from several chronic conditions at the same time, i.e., multimorbidity (Nicholson et al. 2019). However, very little is known of the cumulative effect of experiencing several health conditions simultaneously. If one condition hinders or increases participation, multimorbidity likely has a greater effect than a single condition alone. For example, a higher number of conditions in Finland was previously associated with a lower propensity to vote (Sund et al. 2017). However, less is known about the role of multimorbidity in non-electoral participation forms and other European countries.

## Resources, grievances and age

Building on previous studies (e.g., Söderlund and Rapeli 2015; Sund et al. 2017), we argue that health issues have varying consequences for various forms of political participation. Rather than test hypotheses that address the relationships between individual health issues and specific forms of participation, our approach is more explorative. Our reading of the inconsistencies in the findings of earlier studies suggests that at least three different theoretical approaches might help us understand the linkages between different health issues and forms of participation: *resource*, *grievance* and *social identity theory*. We do not consider these theories as mutually exclusive explanations but as plausible explanations which could help shed light on how health and participation patterns interact.

Resource theory argues that the greater accumulation of various resources increases political participation (Burns et al. 2001; Verba et al. 1995). Resources such as socioeconomic status, time, money and civic skills, together with the ability to acquire, manage and understand political information, allow people to be active in politics more frequently and with less effort (Pacheco 2008; Tam Cho et al. 2006; Verba et al. 1995). Resource theory offers a plausible explanation of the findings reported in most studies, which show that less healthy people have lower levels of political participation, especially turnout. Since health is a type of resource, poorer health decreases turnout. For example, poor health may be connected to low and varying energy levels or decreased income (because of the inability to work), negatively affecting political participation. Furthermore, this demobilizing effect should be stronger for younger people with health issues because they face a “double penalty.” They have fewer resources for participation, both because of their young age and health problems.

Resource theory, however, is less helpful in explaining the findings of studies showing the *reversed health gap*, whereby less healthy individuals report higher levels of some non-electoral forms of political participation. *Grievance theory* (e.g., Gurr 1970; Kern et al. 2015) may be more valid in these cases. Grievances arise based on relative deprivation, where one’s position in the social structure declines relative to one’s reference group. Grievances can be defined as “feelings of dissatisfaction with important aspects of life” (Klandermans et al. 2001: 42). People may perceive relative deprivation when they evaluate their own or their group’s position as disadvantaged compared to other individuals or out-groups (Kelly and Breinlinger 1996; van Zomeren and Iyer 2009; Mattila and Papageorgiou, 2017). In this sense, those relatively disadvantaged may be motivated to participate in public action to improve their situation. Given their declining health, less healthy individuals may thus become more politically engaged to improve their relative position in society. From this perspective, poor health may mobilize individuals into political participation as a motivating force. Research shows that the grievance theory better explains the *positive* impact of poor health on non-institutional participation and the *negative* impact of poor health on institutional political participation (Mattila 2020).

Similarly, *social identity theory* could also explain the reversed health gap in political participation. Social groups form (collective) social identities when they have shared experiences, interests, and solidarities (see, for example, Whooley 2007). Specifically, a collective identity is "an individual's cognitive moral and emotional connection with a broader community, category, practice or institution … [and] … a perception of a shared status or relation …" (Polletta and Jasper 2001: 285). Health is among many factors constituting social identity, e.g., gender, social class, ethnicity, race, or geographical area. For example, health-based identities may form among people experiencing similar health issues and disabilities or any severe health issues in the case of young people. Several health issues are identity-forming, including cancer and diabetes (Park et al. 2009; Thong et al. 2018; Walker and Litchman 2021). Since the expansion of information-communication technology in recent decades, the Internet and social media sites can generate online health communities, e.g., groups with similar health issues. In a study of people living with diabetes, Walker and Litchman (2021) report how their interviewees expressed closeness to other people with diabetes in a diabetes online community. One interviewee said, “*It’s hard to explain, they’re strangers, but they understand parts of me more than my family and friends”*, which was a sentiment “nearly all interviewees expressed.” (p. 920). Taking social identity based on health into account, a motivating force for demonstrating or contacting politicians might not be to secure private (health-related) benefits but to promote collective interests stemming from a group identity among those with health issues. Interestingly, Mattila et al. (2018) found that health-related social identity—measured as feeling solidarity with people who have a long-term illness or a mental health problem—increases only turnout and membership in patient organizations but not other forms of participation, again indicating the need for separate examination of participation varieties.

It is, however, important to recognize that these theories could be simultaneously at play with regard to the link between health and political participation. For example, poorer health might mean a lack of resources for the individual, thus reducing political participation. At the same time, poor health presents a situation where grievances may arise, and health-related social identity is formed, which would increase political participation. Thus, we are not presenting a test of these theories in any strict sense. The question is which of these counteracting forces prevails and in which specific forms of participation. As we believe that the association between health and participation is affected by several simultaneous mechanisms, our aim is not to declare that some of the theories might be wrong but that they may affect peoples’ lives differently depending on their life cycle. Hence, the theories are used to interpret the results our analyses produce. To test these theories in a strict causal sense would require another research design with panel data or some (quasi-)experimental setting.

The link between health conditions and types of participation is also likely to change over a person’s lifespan (see, for example, studies on political participation in older age, Goerres 2007; Bhatti and Hansen 2012; Engelman et al., 2021). However, the few available studies on the potential moderating effect of age have produced mixed results. For example, Mattila et al. (2013) analyzed a sample of 30 European countries and found that poor SRH influenced turnout “mostly among older people”. Among respondents in their 20s or 30s, the differences in voting between those in good health and those in poor health were modest, but the differences became more noticeable among 50-year-olds and older. Pacheco and Fletcher (2015) found that among U.S. adults, the turnout gap between the health groups widened in late adulthood but then slowly narrowed again among the elderly, which suggests that in the U.S., age does moderate the health-participation link. Furthermore, in disability studies, participation gaps among people with disabilities were reported for those over 65 years of age in the case of voting (Schur et al. 2002) and non-voting activities (Schur et al. 2013).

Although the health effect might be smaller among youth than adults and the elderly, poor health also affects young people’s turnout. Ojeda and Pacheco (2019) examined the National Longitudinal Study of Youth panel data for 12–16-year-olds. They found that SRH was associated with lower turnout in one’s first election, but physical limitations were unrelated to voting. Pacheco and Fletcher (2015) analyzed seventh- to twelfth-grade U.S. students surveyed from 1994/95 to 2007/08 and found adolescents with excellent SRH had a subsequent turnout that was seven percentage points higher than those with poor SRH, with the health impact being stronger than, for example, maternal education. In a similar way, Ojeda (2015) showed how depression experienced in adolescence could affect voting and other forms of participation later in life. Depression, for example, may disrupt the formation of a voting habit in young adults leading to lower voting activity in future elections.

While the impact of SRH on voter turnout might increase with age, the opposite might hold true for other forms of political participation. Christensen et al. (2019) found that in Finland, age moderates the association between signing initiatives and SRH. Younger people with poorer health were *more* likely to sign initiatives and petitions than their healthier peers, while there were no such differences among older people with poor and good health.

If health issues were found to have a stable, negative impact on political participation across all life stages, that would signal support for the resource model of participation. However, there are at least two reasons to expect that health affects participation differently at different stages of life. Firstly, people of different ages experience health problems differently. People in their 60s may see a chronic condition as an understandable and acceptable feature of that life stage; in contrast, the same condition in a person in early adulthood can set her apart from her peers and feel very debilitating. From this perspective, compared to their healthier peers, less healthy younger adults may experience more grievances. In short, the relative level of perceived deprivation – the perceived discrepancy between their expectations of life conditions to which they are justifiably entitled, on the one hand, and the degree to which they believe they can obtain these conditions (see Gurr 1970; Kern et al. 2015)—may be greater among young people compared to differences in grievances between less healthy older adults and their healthier peers. Among younger people, those with health problems are more likely to see this as an injustice and may thus feel motivated to work to remedy the situation. They might be particularly likely to opt for more direct forms of participation, e.g., sign petitions or contact public officials to change the health care system or welfare state services. However, it seems plausible that health problems would operate as a greater motivator for younger individuals since they see their situation more in terms of injustice because being sick when older is more expected. In addition, when long-standing health problems arise in early life, health-based identities may be particularly strong, owing to their establishment in the formative period. Social identity could then present additional motivation for increased participation among young people with poor health compared to their peers, resulting in a reversed health gap in political participation among youth.

Thus, we assume that age will moderate the relationship between health and direct forms of political participation so that differences between less healthy and healthier individuals would be greatest among young people and would diminish or even reverse among adults in middle age and the elderly.

Another plausible reason why younger people with health issues may be more likely to participate compared to healthy peers concerns their expected remaining lifetime. Young people, who, despite health problems, can typically be assumed to have more years left than older people, may feel more motivated to participate because they have more at stake, given the probability of living longer. The argument on shorter life expectancy potentially being demotivating for health-beneficial behavior (e.g., healthy lifestyles) is prominent in sociology, epidemiology and economics (see Pampel et al. 2010). In the case of the interplay of health, political participation and age, it could be argued that in comparison with (healthy) elderly adults, less healthy young people have more years to live and, therefore, may be more engaged in political participation with the aim of influencing health policies. Older people, especially those who experience health problems, might be inclined to focus on more immediate goals, including coping with their health issues, but from an individual perspective and not a collective action perspective, which would entail engaging in change through political participation. Again, differences in grievances between unhealthy and healthy older people might be much smaller than among healthy and unhealthy younger people.

However, we also propose a competing assumption. Since young people have fewer other relevant resources such as social networks, integration, and civic skills, their health problems may further raise the bar to participation, making unhealthy young people more politically passive. When the goal is much further away, it could demotivate young people from trying to reach it by being active in public life. In addition, health problems may increasingly motivate individuals in the middle and later stages of life. Middle-aged and older people are more likely to be interested in politics and, because of their frequent health issues, are more dependent on public services. Thus, compared to healthy older adults, experiencing health problems may motivate older adults to engage in politics even more than less healthy younger citizens would be motivated compared to their healthier peers.

## Data and method

We use the 7th round of European Social Survey data (2015), which includes health and political participation data from 20 European countries (see Appendix for more information). Altogether, we have data from about 35,000 respondents across the countries. Since our observations are nested in the respondents’ home countries, we use two-level random-intercept models, where individuals are nested within their countries. The data are also weighted to account for population differences between countries and with post-stratification weights to reduce sampling error and non-response bias (Kaminska 2020).

Our main dependent variables measure political participation. The ESS questionnaire asked if the respondent had taken part in the following seven forms of participation to improve things in their countries during the last 12 months: 1) contacting a politician, government or local government official, 2) working in a political party or action group, 3) working in another organization or association, 4) wearing or displaying a campaign badge/sticker, 5) signing a petition, 6) taking part in a lawful public demonstration, and 7) boycotting certain products. The answer options were Yes and No. Furthermore, respondents’ voting activity was gauged with the question “Did you vote in the last [country] national election in [month/year]?”.

In the following analysis, these forms of participation are analyzed individually and as an index, which sums together all eight participation forms. The general political participation index varies between 0 and 8; its mean is 1.70, and the standard deviation 1.52. The Cronbach’s alpha coefficient for this additive index is 0.63, which is not very high. However, a factor analysis of the same items produces only one factor with an eigenvalue over one.

Individuals’ physical health was measured with 12 items addressing health conditions ranging from cancer to allergies (see Appendix for a list of the conditions and their prevalence in the data). The respondent reported whether they had experienced each condition in the last 12 months. For further analysis, we combined all conditions into a multimorbidity index, which simply counts the number of conditions a person experienced. The multimorbidity index ranges between 0 and 12; its mean is 1.88, and the standard deviation 1.78. People are more likely to experience several simultaneous health conditions as they age. Accordingly, a positive correlation (about 0.3) between age and the multimorbidity index exists.

Since we expect age to moderate the relationship between health conditions and political participation, we must be careful about how it is modeled in the analysis. To ascertain that we had entirely captured the nonlinear relationship, we decided to include age in the models in three forms: as such, in quadratic and cubic forms. However, to keep things relatively simple, when the interactions between health and age are studied, only interactions with the linear age and the specific health condition variables were used.

The relationship between health, age and participation is likely to be confounded by several factors. Hence, we use as controls several variables typically used in studies of political participation (Marien et al. 2010). As socio-demographic controls, we use gender, education (log of years in full-time education), labor market status (employed, unemployed, student, retired or other) and marital status (married or in a civil union, separated or divorced, widowed, never married or in a civil union). Furthermore, we expect that the respondents’ opportunities for engaging in the political activity will be affected by having underage children living at home and by the type of area where they live (big city, suburb of a big city, town or small city, countryside). The likelihood of participation can also be related to economic deprivation, which we tap with a question on how the respondents feel about their current household income (living comfortably, coping, difficult, very difficult). Finally, we also control for the respondents’ birthplace since there might be differences in participation rates between those originally born outside the country where they live and those born in the country.

The complicated data structure, with eight different forms of participation and 12 forms of health conditions, does mean some practical challenges for the analysis. We decided to tackle these problems with various combinations of individual measures of participation and health and index variables formulated from these items. At the beginning of the empirical part, we present results from analyses examining the relationship between specific health conditions and the general political participation index. Then we study how multimorbidity, i.e., the state of experiencing multiple health conditions simultaneously, is related to participation. Since the dependent variable in these analyses is the count of different political participation forms, we use multilevel negative binomial regression as the method, as this method loosens the assumption of the standard Poisson model that the variance is equal to the mean.

Finally, in the last part of the empirical analysis, we study how multimorbidity is associated with individual participation forms, as previous studies have indicated that health problems may be differently manifested through different types of participation (Mattila 2020; Söderlund and Rapeli 2015). Given that the dependent variable here is a dichotomy indicating whether the respondent engaged in a particular participation form, the method used here is multilevel probit regression.

## Results

**Fig. 1:**
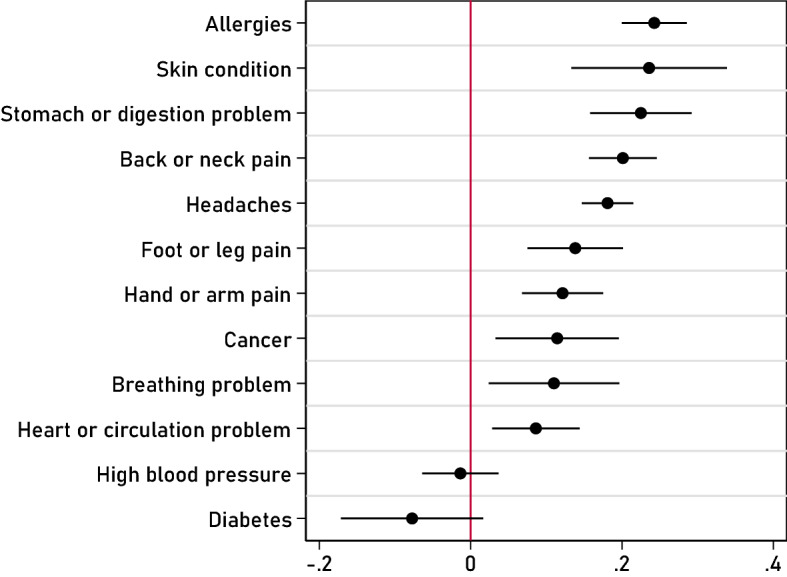
Estimated differences in general political participation between those having a condition and those without the same condition with 95% confidence intervals (estimated from multilevel negative binomial regression modes; the x-axis shows the difference in participation scores between the two groups, positive values indicate that groups with the condition are more active)

We begin by examining whether the physical health conditions included in our analysis are related to the general political participation index. In this first stage, we are interested in the direct relationship between health condition and participation; we do not yet include the interaction between health condition and age in the models. The results for the negative binomial regression analyses are presented visually in Fig. 1 (full results are available in the Appendix). In the analysis, the health conditions are all studied individually, i.e., they are not simultaneously included in the same model. The figure shows the estimated difference in political activity between those with a specific health condition and those without it when all other variables are set to their mean values and the respective 95% confidence intervals.

Figure 1 shows that health conditions, except for high blood pressure and diabetes, have a positive relationship with political participation, meaning that those with the condition are more likely to engage politically than those without the condition. The largest difference is found among those with allergies or skin conditions, who score on average more than 0.2 higher on the participation index than others (the average score for all respondents was 1.70 with a standard deviation of 1.52). This means that the differences are relatively small but still significant. Moreover, the direction of the differences is important: those with health concerns are more engaged in political activity, which aligns with grievance theory expectations: health conditions seem to mobilize people into action.

**Fig. 2:**
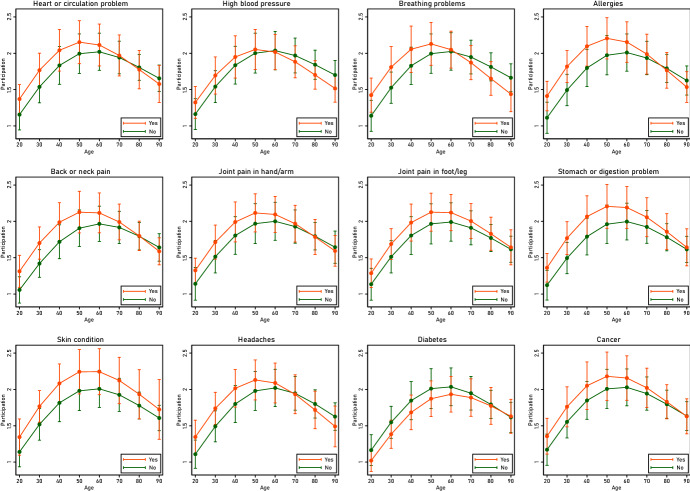
The relationship between physical health conditions and general political participation conditioned by age (negative binomial regression with 95% confidence intervals; participation index ranges between 0 and 8, average = 1.69)

Figure 2 shows results from the models where the interaction terms between the health condition and age are added to see how age moderates the association between health problems and participation. The results are again shown visually for each type of condition. The panels show the estimated values of the general participation index given the person’s age and whether he/she has experienced the particular condition within the last 12 months when controlled for all other factors in the model. Since age is an important factor affecting participation, the curves in each panel follow relatively similar patterns: the level of participation is lower among younger respondents, but participation levels rise until the age of 50 or 60 years, after which participation begins to decline.

The interaction term for age and the health condition is statistically significant for nine of the twelve health conditions. It is not significant (at the 95% level) for back or neck pain, foot or leg pain, and diabetes. It is significant at the *p* < 0.05 level for heart conditions and the *p* < 0.01 level for the rest of the statistically significant conditions. A clear pattern emerges when the interaction term is significant: younger individuals with health conditions are more active than their older counterparts. For most conditions, the differences in activity for those experiencing the condition vs not even out among middle-aged people or a bit older, i.e., among people in their 50s or early 60s. Hence, the positive association between health and participation is moderated by age. This observation is again congruent with the grievance theory. When young people become ill, they may experience it as a greater injustice than an older person might; in the young people’s comparison group, falling ill is less typical than among older people, where it can be considered normal to have some ailment.

**Fig. 3:**
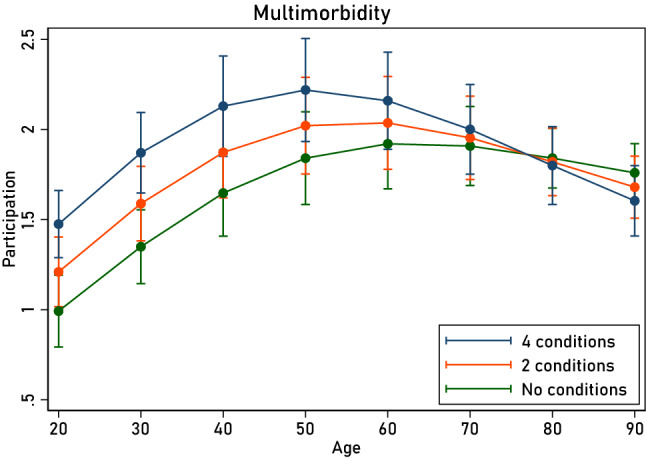
The relationship between multimorbidity and general political participation, conditioned by age (negative binomial regression with 95% confidence intervals; multimorbidity index ranges between 0 and 12, average = 1.87)

This pattern is more clearly depicted in Fig. 3, where we look at the association between general political participation and the index counting the number of conditions each respondent has experienced over the last 12 months. We call this index multimorbidity, which is the co-occurrence of two or more health conditions (Nicholson et al. 2019). The average of this index in our data is 1.87, with a standard deviation of 1.78. At the age of 20, an average person who has not experienced any health conditions scores one on the participation index, while a person with four conditions scores 1.5. This is a relatively large difference in activity, although it must be remembered that multimorbidity is much more common among older citizens. The differences in estimated activity levels between those with four conditions and those with zero conditions lose their significance in the mid-50s. Hence, the mobilizing effect of bad health is mostly effective only among the relatively young.

**Fig. 4:**
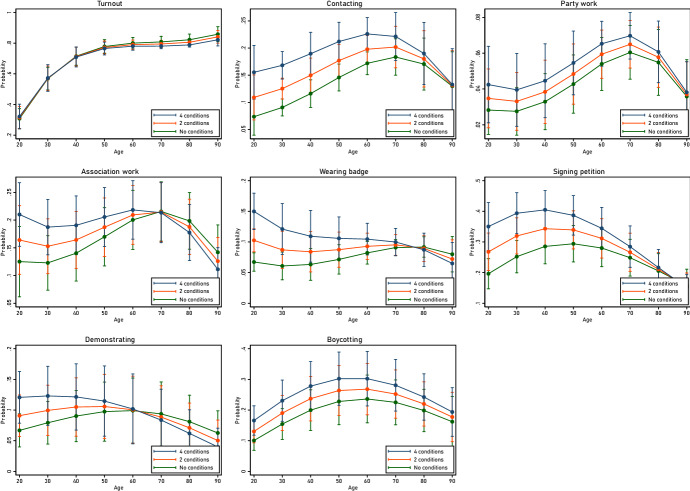
The relationship between multimorbidity and forms of political participation conditioned by age (probit regression with 95% confidence intervals)

Finally, in Fig. 4, we study with multilevel probit analysis how the co-occurrence of multiple health conditions is related to specific forms of participation. At first glance, voting in elections differs from other forms of participation. The estimated difference in turnout between those with zero, two or four conditions is small and statistically insignificant, although it does follow the general patterns found in the literature (Brown et al. 2020), which support the resource theory of political participation by showing that the estimated turnout is higher among healthier people. However, the other forms of participation in Fig. 4 show decidedly different age and health patterns. There are no significant age/condition interactions for working in a party or boycotting certain products for political reasons. However, the interaction is statistically significant for all the other participation forms (*p* < 0.01). For example, the panels for signing petitions, contacting politicians, and wearing badges indicate how younger people with several health conditions are more active than their healthier peers. This suggests that (younger) individuals with health conditions particularly favor non-institutional forms of participation. Additionally, people with health problems are less active in demonstrating and associational work during old age than their healthier counterparts. For these forms of participation, we observe a reversal of the health gap around the age of 65 or 70, suggesting that modes of participation, which may be physically demanding, become less attractive or perhaps practically impossible due to health problems in the later stages of life. Although health problems, broadly speaking, typically seem to motivate participation, it looks as if high age modifies the relationship.

## Discussion and conclusion

The present study examined the interplay between physical health conditions, various forms of political participation, and their varying role over the lifespan. We addressed these three points to advance the current understanding of the relevance of health problems for political participation in European countries. In addition, we examined whether the interplay of the three variables gives more support to resource or grievance theory.

Our results showed that most physical health conditions were related to political participation, but the direction of the effect depends on whether turnout or other forms of participation are analyzed. As reported in previous literature on the link between health and voter turnout (Brown et al. 2020; Burden et al. 2017; e.g., Mattila et al. 2013), we found higher turnout among healthier people, although the differences in our study were not significant when controlling for confounders.

A different picture emerged in our analysis of non-electoral participation. Most physical health conditions we examined mobilized individuals into political action, including non-electoral institutional and non-institutional forms of participation. Our results echo the findings of a Finnish study by Mattila (2020), where better general health was positively related to institutional participation while negatively related to non-institutional participation. In addition, a comparative study of Nordic countries provided similar results (Söderlund and Rapeli 2015). Our results show a similar positive correlation between poor health and non-electoral participation in a broader European context. Furthermore, the condition-specific effects were, by and large, similar according to the various forms of participation examined in our study.

In more detail, we add to the literature by showing that poor physical health benefits non-voting forms of participation up to the age of around 55, with the impact being the strongest in young adulthood. We interpret this as being, at least partly, explained by the timing of the occurrence of an illness, which may affect a person’s view of their illness and self-image. For example, developing a chronic illness at a vulnerable point in adolescence or young adulthood could profoundly affect political participation through social identity development and a sense of belonging. In this view, younger persons affected by health issues are more likely to develop a group identity, especially with the help of civil society organizations, concerning chronic conditions, which may lead to more participation. There are several reasons why this might be the case. First, when older people were younger, health issues were more stigmatized than now. Hence, it was less likely that they developed a strong health-related group identity in their youth. Second, older people are less likely to develop a new identity when they get ill because they are less likely to change their identities, which tend to be formed early in life. Third, identity development nowadays also happens online, and younger people are more apt to use the Internet and social media, which may help generate health-based social identities.

In contrast, developing health issues later in life often leads to a decreased ability to continue working and interferes with social life (Mattila et al. 2018), which may result in a strong demobilizing effect (e.g., Verba et al. 1995). In addition, developing a social identity later in life because of health issues might be more complicated than in young adulthood, all of which point to the demobilizing effect of poor health in late adulthood. As mentioned, young people may also find health conditions especially unjust since most of their peers are in good health, which may propel less healthy young individuals toward political action, in contrast to less healthy older adults.

Hence, the interpretation of the results can be quite complex on a more general level. Participation may be simultaneously linked to all three theories, but the importance of these theories is different depending on a person’s age. The grievance and identity theories are more relevant when discussing the health gap among younger people. In contrast, the resource theory better explains the waning political activity among older citizens. This is especially due to multimorbidity, which is more likely among senior citizens.

Since the measures available in our studies did not provide information on when the physical health condition(s) began, future studies should examine how the life-stage timing of health problems and their length of presence might explain the moderating role of age in the link between health and participation. It is possible that the variable age in our analyses also captures the effects of the maturation of the illness, possibly acquired years ago. However, to analyze such a hypothesis, panel data are required. Furthermore, subsequent research could, for example, investigate mediating mechanisms such as group belonging. Existing evidence on mental health suggests that a lengthy accumulation of mental health symptoms may have an increasingly negative impact on psychological political engagement (e.g., external political efficacy; Bernardi et al. 2022), which is an important group of predictors of political participation (Kirbiš et al. 2017; Verba et al. 1995).

Besides specific health conditions, our results suggest that multimorbidity is also important since the more physical health conditions a person suffers from, the more he/she is likely to be politically active, especially when young. This is again consistent with grievance theory since, in our study, the accumulation of conditions (i.e., a proxy for potential grievances) increased participation. Our findings echo a study by Mattila and Papageorgiou (2017), who found that in European countries, disability decreased turnout, especially when coupled with grievances (as measured by perceived discrimination); however, it increased demonstrating and contacting politicians (also see Reher 2020). Turnout might be decreased among those with poor health since individuals who identify with a broad community based on their health condition feel alienated by a political system, which does not seem to prioritize the group’s concerns and where not many representatives openly identify with their social group. In contrast, the multimorbidity and participation link detected in our study is inconsistent with resource theory, except for voting. Although beyond the purpose of our study, we may speculate on the mechanisms explaining the link between poorer health and increased non-voting political participation. Political trust, for example, has previously been implicated in the link between poor health and non-institutional participation (e.g., Mattila 2020); because less healthy people report lower levels of political trust, they are more likely to turn to non-institutional participation. In addition, the mobilization potential of poor health may also be related to increased interest in health-related policy matters (Mattila et al. 2020; Söderlund and Rapeli 2015).

We also found differences in the impact of various health conditions on the participation index. However, it is not possible to give a straightforward interpretation of why differences are larger among people with certain conditions than among those with other conditions. One problem is distinguishing the conditions' severity from the survey data. For example, allergies or skin conditions can be mild or so severe that they considerably restrict participation in everyday activities. Hence, it is only possible to say that most health conditions examined in our study are associated with increased political participation. As previously argued by others (e.g., Christensen et al. 2019), future studies should consider the severity of specific health conditions to examine their impact on participation.

Some limitations of the present study should be mentioned. Since we employed a cross-sectional study design, causal mechanisms could not be addressed. Longitudinal panel designs are needed to ascertain the causal mechanisms explaining the health and participation link. For example, among the potential mediators explaining increased participation among the less healthy, perceived grievance, social identity, social integration, decreased political trust, and external political efficacy could be examined in future studies. Second, our study examined physical health conditions, but future research should also examine other health indicators, especially mental health issues. In addition, since the association between health and participation depends on other social status factors, including race and SES (e.g., Gollust and Rahn 2015), scholars should also investigate whether the moderating role of age differs among various social groups. It should also be mentioned that the effect of age could be due to a generation effect, so future longitudinal studies should also examine this possibility.

Notwithstanding these limitations, the present study extends our knowledge of how health inequalities translate into inequalities in political participation. These, in turn, have important implications for the functioning and legitimacy of contemporary democracies. It has previously been documented that citizens’ policy preferences differ significantly across their health status, and political representatives are unevenly responsive to their constituents according to constituents’ health status (Ojeda and Pacheco 2019). Our study suggests that *some* specific health conditions may lead to participation inequalities and inequalities in representation, while *others* may motivate and mobilize constituents. Understanding the determinants and mechanisms of unequal political participation in democratic systems can provide a basis for effective policies to address and diminish democratic inequalities.

Our study also suggests that physical health conditions increase non-voting political participation at earlier life stages and up to the mid-50s. Less healthy people tend to have fewer available resources (Adler and Newman 2002; Mackenbach et al. 2008) and lower resources decrease political participation (Verba et al. 1995). However, health conditions mobilizing (young) adult Europeans is an encouraging result, particularly from the point of view of normative political theory (see Teorell 2006) and for reducing health inequalities through public policy (Ojeda and Pacheco 2019). Although less healthy people seem to have a stronger voice in non-voting participation, it remains unknown whether this compensates for their generally lower turnout and consequent differences in policy outcomes because voting remains the most common form of political participation, with the most direct consequences for party choice and public policy. Nonetheless, our study suggests that certain forms of political participation provide channels for political engagement among the less healthy public, especially young adults.

The more general lesson to be drawn from the analysis concerns the fundamental drivers of individual political behavior. Although there is a long-standing tradition in the political behavior literature of considering various kinds of disturbance as obstacles to participation, our findings demonstrate that inconvenience can instead be a motivator for participation. Arguably, health issues are a personal matter, which also makes them rather serious from the affected individual's perspective. Consequently, obstacles to participation seem to be drivers of participation when the stakes are high enough for the individual. Given the prominent status of the resource theory approach in the field, we encourage scholars to challenge the conventional view that focuses on the lack of resources as inherently detrimental to citizen engagement.

1. We repeated the models with a reduced set of control variables as a sensitivity test. However, the results remained similar when only gender, age and education were used as controls.

## Figures and Tables

**Fig. 5:**
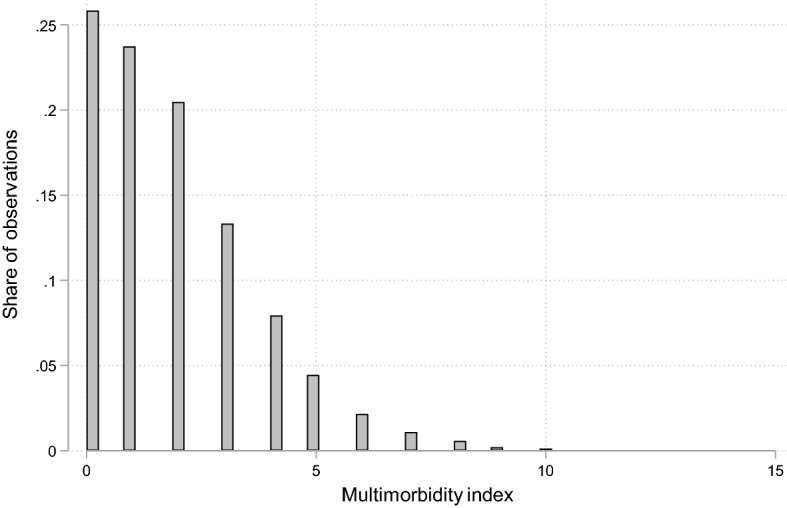
Distribution of the multimorbidity index

**Fig. 6:**
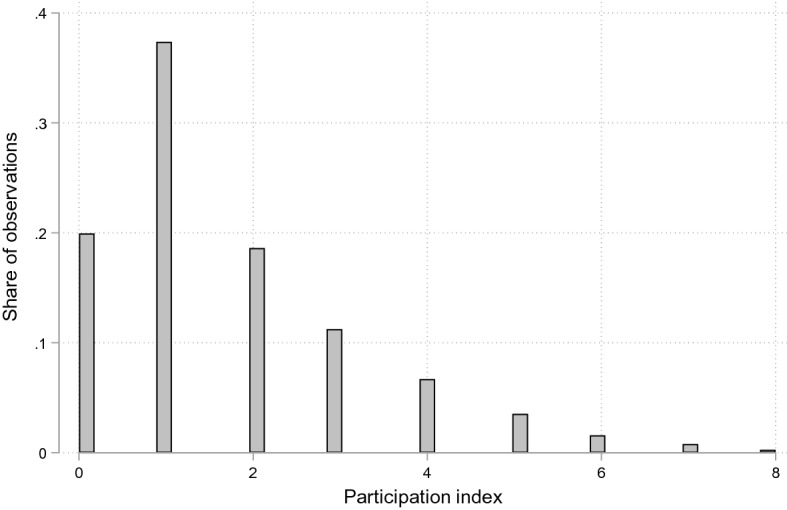
Distribution of the participation index

## Appendix

See Figs. 5, 6 and Tables

**Table 1:** Prevalence of physical conditions within the last 12 months

	Mean	Standard deviation	Minimum value	Maximum value
Heart or circulation problem	0.104	0.306	0	1
High blood pressure	0.184	0.387	0	1
Breathing problems	0.084	0.278	0	1
Allergies	0.116	0.320	0	1
Back or neck pain	0.375	0.484	0	1
Muscular or joint pain in the hand or arm	0.207	0.405	0	1
Muscular or joint pain in the foot or leg	0.230	0.421	0	1
Stomach or digestion related	0.151	0.358	0	1
Skin condition related	0.082	0.274	0	1
Severe headaches	0.129	0.335	0	1
Diabetes	0.057	0.232	0	1
Cancer*	0.115	0.318	0	1
Multimorbidity index	1.877	1.779	0	12

*Cancer, leukaemia, malignant tumor, malignant lymphoma, melanoma, carcinoma or other skin cancer currently or previously suffered

1,

**Table 2:** Political participation within the last 12 months

	Mean	Standard deviation	Minimum value	Maximum value
Turnout	0.699	0.459	0	1
Contact politicians	0.161	0.367	0	1
Working in a party	0.045	0.207	0	1
Working in an association	0.174	0.379	0	1
Wearing badge	0.087	0.282	0	1
Signing petitions	0.250	0.433	0	1
Participation in demonstrations	0.074	0.261	0	1
Boycotting	0.195	0.396	0	1
Participation index	1.698	1.524	0	8

^2^,

**Table 3:** The effect of health conditions, age and their interaction on political participation (*N* = 34,913)

	Heart / circulation problem	High blood pressure	Breathing problems	Allergies	Back or neck pain	Muscular or joint pain in the hand or arm
Condition	0.232**	0.200**	0.328**	0.322**	0.288**	0.202
	(0.071)	(0.063)	(0.086)	(0.050)	(0.020)	(0.112)
Condition * Age	−0.031*	−0.035**	−0.053**	−0.042**	−0.036**	−0.026
	(0.013)	(0.010)	(0.015)	(0.010)	(0.003)	(0.019)
Age	0.644**	0.629**	0.657**	0.647**	0.624**	0.630**
	(0.189)	(0.187)	(0.191)	(0.196)	(0.182)	(0.185)
Age * Age	−0.084*	−0.081*	−0.085*	−0.082*	−0.076*	−0.081*
	(0.035)	(0.035)	(0.035)	(0.036)	(0.034)	(0.034)
Age * Age * Age	0.003	0.003	0.003	0.003	0.003	0.003
	(0.002)	(0.002)	(0.002)	(0.002)	(0.002)	(0.002)
Female	−0.034**	−0.032**	−0.034**	−0.037**	−0.041**	−0.034**
	(0.010)	(0.009)	(0.009)	(0.008)	(0.011)	(0.010)
Education	0.725**	0.725**	0.723**	0.716**	0.717**	0.726**
	(0.064)	(0.063)	(0.064)	(0.062)	(0.065)	(0.065)
Divorced	−0.055**	−0.056**	−0.055**	−0.057**	−0.055**	−0.057**
	(0.017)	(0.018)	(0.018)	(0.019)	(0.017)	(0.018)
Widow	−0.067**	−0.065**	−0.064**	−0.067**	−0.064**	−0.067**
	(0.023)	(0.022)	(0.023)	(0.023)	(0.023)	(0.023)
Never married	−0.039	−0.040	−0.040	−0.041	−0.036	−0.040
	(0.038)	(0.038)	(0.039)	(0.038)	(0.035)	(0.038)
Studying	0.071*	0.070*	0.068*	0.062*	0.082**	0.072*
	(0.029)	(0.029)	(0.029)	(0.027)	(0.032)	(0.028)
Unemployed	−0.096**	−0.096**	−0.098**	−0.095**	−0.093*	−0.095**
	(0.036)	(0.036)	(0.037)	(0.036)	(0.036)	(0.035)
Retired	0.007	0.013	0.012	0.009	0.009	0.010
	(0.029)	(0.030)	(0.029)	(0.029)	(0.029)	(0.029)
Other	−0.052	−0.048	−0.049	−0.050	−0.051	−0.051
	(0.028)	(0.028)	(0.028)	(0.028)	(0.028)	(0.029)
Children at home	−0.028	−0.030	−0.028	−0.028	−0.032	−0.030
	(0.023)	(0.024)	(0.023)	(0.024)	(0.024)	(0.024)
Living in suburbs of big city	−0.068**	−0.069**	−0.070**	−0.067**	−0.068**	−0.067**
	(0.020)	(0.019)	(0.020)	(0.020)	(0.020)	(0.019)
Living in small city	−0.092**	−0.091**	−0.092**	−0.089**	−0.092**	−0.092**
	(0.027)	(0.027)	(0.028)	(0.027)	(0.026)	(0.027)
Living in village	−0.087*	−0.087*	−0.088*	−0.084*	−0.086*	−0.087*
	(0.037)	(0.038)	(0.039)	(0.039)	(0.036)	(0.038)
Living in countryside	−0.073*	−0.074**	−0.073*	−0.068*	−0.077**	−0.074*
	(0.029)	(0.029)	(0.029)	(0.028)	(0.028)	(0.029)
Coping on present income	−0.077**	−0.076**	−0.077**	−0.075**	−0.080**	−0.079**
	(0.014)	(0.013)	(0.014)	(0.013)	(0.013)	(0.013)
Difficult on present income	−0.138**	−0.135**	−0.139**	−0.135**	−0.144**	−0.140**
	(0.018)	(0.018)	(0.018)	(0.018)	(0.019)	(0.018)
Very difficult on present income	−0.197**	−0.191**	−0.199**	−0.191**	−0.206**	−0.201**
	(0.057)	(0.056)	(0.057)	(0.057)	(0.055)	(0.057)
Not born in country	−0.314**	−0.315**	−0.313**	−0.312**	−0.303**	−0.315**
	(0.063)	(0.063)	(0.063)	(0.063)	(0.064)	(0.063)
Intercept	−2.501**	−2.477**	−2.532**	−2.529**	−2.553**	−2.498**
	(0.277)	(0.273)	(0.280)	(0.289)	(0.261)	(0.280)
Country level variance	0.060	0.059	0.058	0.058	0.054	0.058
	(0.024)	(0.023)	(0.023)	(0.023)	(0.022)	(0.023)

	Muscular or joint pain in the foot or leg	Stomach or digestion related	Skin condition	Headaches	Diabetes	Cancer
Condition	0.156**	0.244**	0.191**	0.273**	−0.173	0.189**
	(0.056)	(0.046)	(0.059)	(0.066)	(0.132)	(0.048)
Condition * Age	−0.016	−0.025**	−0.013	−0.040**	0.020	−0.021**
	(0.010)	(0.007)	(0.014)	(0.014)	(0.021)	(0.008)
Age	0.644**	0.626**	0.633**	0.644**	0.629**	0.636**
	(0.187)	(0.180)	(0.189)	(0.176)	(0.189)	(0.190)
Age * Age	−0.083*	−0.078*	−0.081*	−0.080*	−0.080*	−0.082*
	(0.034)	(0.034)	(0.035)	(0.033)	(0.035)	(0.035)
Age * Age * Age	0.003	0.003	0.003	0.003	0.003	0.003
	(0.002)	(0.002)	(0.002)	(0.002)	(0.002)	(0.002)
Female	−0.032**	−0.039**	−0.036**	−0.044**	−0.034**	−0.034**
	(0.009)	(0.010)	(0.009)	(0.009)	(0.010)	(0.009)
Education	0.725**	0.716**	0.719**	0.722**	0.723**	0.722**
	(0.064)	(0.064)	(0.063)	(0.064)	(0.063)	(0.063)
Divorced	−0.057**	−0.060**	−0.056**	−0.056**	−0.055**	−0.059**
	(0.018)	(0.018)	(0.018)	(0.017)	(0.018)	(0.018)
Widow	−0.068**	−0.067**	−0.064**	−0.064**	−0.067**	−0.069**
	(0.024)	(0.024)	(0.023)	(0.023)	(0.024)	(0.024)
Never married	−0.040	−0.042	−0.039	−0.040	−0.039	−0.041
	(0.038)	(0.038)	(0.037)	(0.039)	(0.038)	(0.038)
Studying	0.068*	0.066*	0.069*	0.074*	0.070*	0.075**
	(0.028)	(0.030)	(0.028)	(0.031)	(0.029)	(0.028)
Unemployed	−0.094**	−0.101**	−0.094**	−0.099**	−0.096**	−0.100**
	(0.035)	(0.036)	(0.035)	(0.034)	(0.036)	(0.037)
Retired	0.009	0.006	0.009	0.007	0.012	0.008
	(0.028)	(0.028)	(0.030)	(0.030)	(0.029)	(0.029)
Other	−0.051	−0.054	−0.050	−0.052	−0.046	−0.051
	(0.030)	(0.029)	(0.027)	(0.029)	(0.028)	(0.028)
Children at home	−0.028	−0.026	−0.029	−0.032	−0.029	−0.031
	(0.023)	(0.023)	(0.024)	(0.024)	(0.024)	(0.024)
Living in suburbs of big city	−0.068**	−0.069**	−0.066**	−0.067**	−0.068**	−0.067**
	(0.020)	(0.020)	(0.020)	(0.020)	(0.020)	(0.020)
Living in small city	−0.091**	−0.088**	−0.089**	−0.091**	−0.091**	−0.089**
	(0.027)	(0.027)	(0.027)	(0.027)	(0.027)	(0.027)
Living in village	−0.085*	−0.082*	−0.083*	−0.087*	−0.087*	−0.086*
	(0.037)	(0.037)	(0.039)	(0.038)	(0.038)	(0.038)
Living in countryside	−0.072*	−0.071**	−0.068*	−0.074*	−0.073*	−0.071*
	(0.030)	(0.027)	(0.028)	(0.030)	(0.029)	(0.029)
Coping on present income	−0.079**	−0.077**	−0.076**	−0.078**	−0.076**	−0.075**
	(0.013)	(0.014)	(0.013)	(0.013)	(0.013)	(0.013)
Difficult on present income	−0.142**	−0.138**	−0.139**	−0.142**	−0.134**	−0.130**
	(0.018)	(0.018)	(0.017)	(0.018)	(0.017)	(0.017)
Very difficult on present income	−0.202**	−0.198**	−0.196**	−0.199**	−0.189**	−0.193**
	(0.056)	(0.059)	(0.058)	(0.057)	(0.056)	(0.058)
Not born in the country	−0.311**	−0.311**	−0.311**	−0.314**	−0.315**	−0.317**
	(0.063)	(0.064)	(0.062)	(0.062)	(0.062)	(0.063)
Intercept	−2.520**	−2.487**	−2.491**	−2.538**	−2.476**	−2.470**
	(0.280)	(0.261)	(0.272)	(0.251)	(0.276)	(0.276)
Country level variance	0.058	0.058	0.058	0.058	0.060	0.057
	(0.023)	(0.023)	(0.023)	(0.023)	(0.023)	(0.025)

3,

**Table 4:** The effect of multimorbidity on political participation (*N* = 32,857)

Multimorbidity index	0.134**
	(0.019)
Multimorbidity * Age interaction	−0.017**
	(0.003)
Age	0.663**
	(0.176)
Age * Age	−0.084**
	(0.032)
Age * Age * Age	0.003
	(0.002)
Female	−0.048**
	(0.009)
Education	0.712**
	(0.065)
Studying	0.070*
	(0.029)
Unemployed	−0.104**
	(0.035)
Retired	−0.002
	(0.029)
Other	−0.072*
	(0.031)
Children at home	−0.032
	(0.025)
Living in suburbs of big city	−0.066**
	(0.021)
Living in small city	−0.086**
	(0.028)
Living in village	−0.081*
	(0.038)
Living in countryside	−0.068*
	(0.029)
Coping on present income	−0.082**
	(0.013)
Difficult on present income	−0.151**
	(0.019)
Very difficult on present income	−0.231**
	(0.063)
Not born in country	−0.301**
	(0.064)
Divorced	−0.064**
	(0.020)
Widow	−0.065**
	(0.023)
Never married	−0.044
	(0.038)
Intercept	−2.640**
	(0.261)
Country level variance	0.050
	(0.023)

4,

**Table 5:** The effect of multimorbidity on participation forms

	Turnout	Contacting politicians	Working in a party	Working in an association
Multimorbidity index	0.026	0.146**	0.061*	0.136**
	(0.018)	(0.018)	(0.026)	(0.028)
Multimorbidity * Age interaction	−0.008	−0.016**	−0.006	−0.020**
	(0.004)	(0.003)	(0.005)	(0.005)
Age	1.893**	−0.099	−0.473	−0.525
	(0.212)	(0.470)	(0.289)	(0.295)
Age * Age	−0.280**	0.064	0.125*	0.139*
	(0.046)	(0.088)	(0.055)	(0.055)
Age * Age * Age	0.014**	−0.005	−0.009**	−0.010**
	(0.003)	(0.005)	(0.003)	(0.003)
Female	−0.011	−0.204**	−0.251**	−0.150**
	(0.020)	(0.028)	(0.050)	(0.022)
Education	0.652**	0.680**	0.749**	0.865**
	(0.141)	(0.066)	(0.101)	(0.073)
Studying	−0.248**	0.047	0.073	0.195**
	(0.088)	(0.042)	(0.076)	(0.051)
Unemployed	−0.253**	−0.063*	0.068	−0.171**
	(0.050)	(0.031)	(0.086)	(0.038)
Retired	0.168**	−0.111**	−0.047	−0.057
	(0.063)	(0.032)	(0.098)	(0.072)
Other	−0.080**	−0.076	0.086	−0.106**
	(0.029)	(0.048)	(0.061)	(0.036)
Children at home	−0.091**	0.067	0.021	0.025
	(0.032)	(0.048)	(0.031)	(0.035)
Living in suburbs of big city	0.004	0.024	−0.101**	−0.051
	(0.024)	(0.025)	(0.032)	(0.034)
Living in small city	−0.034	0.042	−0.087	0.020
	(0.025)	(0.066)	(0.049)	(0.061)
Living in village	0.010	0.173*	−0.096	0.023
	(0.026)	(0.076)	(0.084)	(0.089)
Living in countryside	−0.030	0.147*	0.021	0.105
	(0.044)	(0.063)	(0.090)	(0.088)
Coping on present income	−0.131**	−0.083*	−0.050	−0.159**
	(0.021)	(0.035)	(0.040)	(0.011)
Difficult on present income	−0.325**	−0.105*	−0.104**	−0.249**
	(0.033)	(0.053)	(0.032)	(0.059)
Very difficult on present income	−0.440**	−0.026	−0.169	−0.301**
	(0.039)	(0.092)	(0.105)	(0.094)
Not born in country	−0.956**	−0.120	−0.063	−0.071
	(0.122)	(0.066)	(0.103)	(0.045)
Divorced	−0.353**	−0.052	−0.016	−0.083**
	(0.021)	(0.049)	(0.069)	(0.024)
Widow	−0.187**	−0.052	−0.083	0.015
	(0.053)	(0.052)	(0.115)	(0.061)
Never married	−0.132**	−0.164**	−0.057	−0.078
	(0.040)	(0.038)	(0.064)	(0.045)
Intercept	−4.616**	−3.084**	−3.137**	−2.666**
	(0.233)	(0.660)	(0.510)	(0.452)
Country level variance	0.082	0.022	0.048	0.147
	(0.026)	(0.011)	(0.018)	(0.035)
*N*	34,299	34,505	34,507	33,329

	Wearing a badge	Signing petitions	Demonstrations	Boycotting
Multimorbidity index	0.165**	0.169**	0.135**	0.102**
	(0.023)	(0.027)	(0.016)	(0.027)
Multimorbidity * Age interaction	−0.022**	−0.020**	−0.022**	−0.007
	(0.004)	(0.005)	(0.003)	(0.004)
Age	−0.388	0.490**	0.121	0.573**
	(0.270)	(0.091)	(0.210)	(0.145)
Age * Age	0.087	−0.064**	0.001	−0.061
	(0.053)	(0.019)	(0.050)	(0.031)
Age * Age * Age	−0.005	0.002	−0.001	0.001
	(0.003)	(0.001)	(0.003)	(0.002)
Female	−0.002	0.027	−0.095**	0.059*
	(0.032)	(0.016)	(0.009)	(0.030)
Education	0.552**	0.896**	0.576**	0.795**
	(0.069)	(0.058)	(0.042)	(0.062)
Studying	0.114*	0.082	0.128*	0.158*
	(0.057)	(0.053)	(0.051)	(0.064)
Unemployed	−0.077	−0.081	−0.003	0.011
	(0.044)	(0.056)	(0.070)	(0.038)
Retired	−0.167**	0.039	−0.110*	−0.045
	(0.046)	(0.069)	(0.048)	(0.076)
Other	−0.124*	−0.039	−0.149*	−0.045
	(0.062)	(0.037)	(0.072)	(0.028)
Children at home	−0.093**	−0.020	−0.060	−0.037
	(0.015)	(0.031)	(0.036)	(0.031)
Living in suburbs of big city	−0.098**	−0.018	−0.306**	−0.111*
	(0.035)	(0.037)	(0.046)	(0.056)
Living in small city	−0.107**	−0.120**	−0.311**	−0.180**
	(0.040)	(0.026)	(0.061)	(0.060)
Living in village	−0.127**	−0.144**	−0.483**	−0.225*
	(0.037)	(0.045)	(0.047)	(0.090)
Living in countryside	−0.191*	−0.188**	−0.511**	−0.074
	(0.082)	(0.048)	(0.081)	(0.089)
Coping on present income	−0.091**	−0.060	−0.038	−0.109**
	(0.025)	(0.041)	(0.028)	(0.042)
Difficult on present income	−0.060	−0.172**	−0.032	−0.057
	(0.056)	(0.038)	(0.023)	(0.041)
Very difficult on present income	−0.181*	−0.206*	−0.090	−0.167
	(0.081)	(0.083)	(0.055)	(0.106)
Not born in country	−0.123	−0.287**	0.040	−0.131**
	(0.085)	(0.091)	(0.093)	(0.047)
Divorced	0.108	0.034	0.096	−0.067**
	(0.081)	(0.042)	(0.063)	(0.022)
Widow	−0.001	−0.089	−0.176	−0.139**
	(0.071)	(0.050)	(0.098)	(0.054)
Never married	−0.078	0.004	0.078	0.038
	(0.086)	(0.049)	(0.099)	(0.043)
Intercept	−2.243**	−3.760**	−2.942**	−4.147**
	(0.209)	(0.247)	(0.234)	(0.216)
Country level variance	0.079	0.108	0.150	0.192
	(0.025)	(0.041)	(0.062)	(0.061)
*N*	34,506	34,448	34,514	34,428

^5^.
